# Preventive effects of *Morus alba L.* anthocyanins on diabetes in Zucker diabetic fatty rats

**DOI:** 10.3892/etm.2013.1203

**Published:** 2013-07-04

**Authors:** ARIYA SARIKAPHUTI, THAMTHIWAT NARARATWANCHAI, TERUTO HASHIGUCHI, TAKASHI ITO, SITA THAWORANUNTA, KIYOSHI KIKUCHI, YOKO OYAMA, IKURO MARUYAMA, SALUNYA TANCHAROEN

**Affiliations:** 1 School of Anti-Aging and Regenerative Medicine, Mae Fah Luang University, Bangkok 10110, Thailand; 2 Department of Laboratory and Vascular Medicine, Kagoshima University Graduate School of Medical and Dental Sciences, Kagoshima 890-8520, Japan; 3 Department of Systems Biology in Thromboregulation, Kagoshima University Graduate School of Medical and Dental Sciences, Kagoshima 890-8520, Japan; 4 Department of Prosthodontics, Faculty of Dentistry, Mahidol University, Bangkok 10400, Thailand; 5 Departments of Physiology and Neurosurgery, Kurume University School of Medicine, Fukuoka 830-0011, Japan; 6 Department of Pharmacology, Faculty of Dentistry, Mahidol University, Bangkok 10400, Thailand

**Keywords:** *Morus alba L*, anthocyanins, type 2 diabetes, disease prevention

## Abstract

The mulberry plant (*Morus alba L.*) contains abundant anthocyanins (ANCs), which are natural antioxidants. The aim of this study was to determine the ANC composition of Thai *Morus alba* L. fruits and to assess the effect of an ANC extract on blood glucose and insulin levels in male leptin receptor-deficient Zucker diabetic fatty (ZDF) rats. The major components of the ANC extract were identified by high-performance liquid chromatography-electrospray ionization-mass spectrometry. ZDF and lean rats were treated with 125 or 250 mg ANCs/kg body weight, or 1% carboxymethylcellulose (CMC) twice daily for 5 weeks. Neither ANC dose had an effect on body weight. Following 5 weeks of treatment, glucose levels were observed to increase from 105.5±8.7 to 396.25±21 mg/dl (P<0.0001) in the CMC-treated ZDF rats; however, the glucose levels were significantly lower in the rats treated with 125 or 250 mg/kg ANCs (228.25±45 and 131.75±10 mg/dl, respectively; P<0.001 versus CMC). The administration of 250 mg/kg ANCs normalized glucose levels in the ZDF rats towards those of the lean littermates. Insulin levels were decreased significantly in the ZDF rats treated with CMC or 125 mg/kg ANCs (P<0.0001), but not in the rats treated with 250 mg/kg ANCs. Histologically, 250 mg/kg ANCs was observed to prevent islet degeneration compared with the islets in CMC-treated rats. This study, demonstrated that ANCs extracted from *Morus alba L.* were well tolerated and exhibited effective anti-diabetic properties in ZDF rats. ANCs represent a promising class of therapeutic compounds that may be useful in the prevention of type 2 diabetes.

## Introduction

Type 2 diabetes is preceded by the inability of β-cells to secrete sufficient insulin to overcome insulin resistance or reduced insulin sensitivity, combined with reduced insulin secretion. Degeneration of the islets of Langerhans with β-cell loss is secondary to insulin resistance and is regarded as the pathophysiology of type 2 diabetes (1). Oral hypoglycemic agents directly stimulate insulin release from β-cells to overcome insulin resistance and normalize blood glucose levels. However, these drugs may induce certain adverse effects, such as hypoglycemia (2,3). The consumption of anthocyanins (ANCs) has been suggested to be correlated with a reduced risk of degenerative diseases, such as atherosclerosis (4), cardiovascular diseases (5), cancer (6) and diabetes (7). ANCs extracted from *Calendula officinalis* fruits have been reported to enhance insulin release from pancreatic β-cells *in vitro*(8). Mulberry leaves and fruits have been used in the treatment of numerous diseases (9–12). The mulberry fruit (*Morus alba L*., family Moraceae) contains abundant ANCs, which scavenge reactive oxygen species (13), have anti-obesity effects and inhibit low-density lipoprotein oxidation (14). The predominant ANCs in mulberry, cyanidin 3-rutinoside and cyanidin 3-glucoside, have been demonstrated to dose-dependently inhibit the migration and invasion of highly metastatic A549 human lung carcinoma cells (15). Furthermore, it was recently demonstrated that the cyanidin 3-O-β-D-glucopyranoside fraction from mulberry fruit protected against bladder dysfunction in streptozotocin-induced diabetic rats (16). However, it has not yet been elucidated whether the ANCs in mulberry are able to significantly lower blood glucose levels and whether they may be useful in the treatment of the pathogenesis of type 2 diabetes.

The evolution of diabetes in male leptin receptor-deficient Zucker diabetic fatty (ZDF) rats (ZDF/CrlCrlj) has resulted in it becoming a popular model for preclinical studies of type 2 diabetes, due to the fact that these rats exhibit disrupted islet architecture, β-cell degranulation and increased β-cell death (17,18). Therefore, ZDF male rats were used as a rodent model of type 2 diabetes in the present study. It was hypothesized that the consumption of an ANC extract from Thai *Morus alba L.* fruits was likely to result in glucose-lowering effects and enhanced insulin secretion. The purpose of this study was to determine the ANC composition of Thai *Morus alba L.* fruits, and to assess the effect of an ANC extract on the blood glucose and insulin levels in ZDF rats. To the best of our knowledge, the present study has demonstrated for the first time that ANCs extracted from Thai *Morus alba L.* have significant anti-diabetic activity. Furthermore, the ANC extract appeared to prevent the development of pathogenic lesions in diabetic islets by suppressing islet degeneration.

## Material and methods

### Plant material and extraction

Mulberry fruits were obtained from Kamnan Jul Farm, Petchaboon Province, Thailand. The fruit was extracted in ethanol-water (50/50, v/v%), prior to the extract being filtered through a Buchner funnel and filter paper (Chmlab, Barcelona, Spain) and transferred to a 100 ml flask. The extract was then collected and condensed at 40°C using a Büchi B-490 rotary evaporator (Büchi Labortechnik AG, Flawil, Switzerland) under a vacuum and lyophilized with a freeze-dryer (Labconco Corp., Kansas City, MO, USA).

### Isolation and purification of mulberry ANCs

A C18 Sep-Pak cartridge (Waters Corp., Milford, MO, US) was activated for 30 min with distilled water and high-performance liquid chromatography (HPLC)-grade methanol (Merck KGaA, Darmstadt, Germany). The ANC extract was then loaded onto the column. Following successive washes with five volumes of distilled water (acidified with 0.01% HCl) and ethyl acetate (Fisher Scientific UK Ltd., Loughborough, UK), the ANCs were eluted with methanol containing 0.01% HCl. The ANC solution was then collected and condensed at 40°C using a Büchi B-490 rotary evaporator under vacuum.

### HPLC-electrospray ionization (ESI)-mass spectrometry (MS)

ANCs in the partially purified extracts were separated and quantified by reverse-phase HPLC using a Hypersil™ Gold C18 column (inner diameter, 5 μm; 4.6×250 mm; Thermo Fisher Scientific Inc., Salt Lake City, IL, USA). The column was eluted with a mobile phase consisting of water, 3.75% formic acid (VWR International, Ltd., Lutterworth, UK) and 15% methanol at a flow rate of 1 ml/min. The separated ANCs were detected and measured at 530 nm, and were identified based on the retention times and ultraviolet (UV)-visible (Vis) wavelength spectra of pure authentic standards (cyanidin 3-O-glucoside, cyanidin 3-rutinoside, pelargonidin 3-glucoside and pelargonidin 3-rutinoside; Sigma, St. Louis, MO, USA). The identity of each peak was verified by LC-MS (Agilent 1100; Agilent Technologies, Santa Clara, CA, USA) using ESI and operating in a single quadrupole mode. The instrument was scanned over the *m/z* range of 200–1,500 in the ESI positive ion mode. The LC-MS was eluted with acetonitrile (Fisher Scientific UK Ltd.) and 0.5% ammonium hydroxide (90:10, v/v%).

### Quantification of ANCs by UV-Vis spectroscopy

The ANCs were quantified by UV-Vis spectroscopy, as previously described (19). The model reaction solution was diluted with 0.01% HCl in distilled water and the absorbance at 510 nm was compared with that of known standard solutions using a Genesys 10 UV spectrophotometer (Thermo Spectronic, Rochester, NY, USA).

### Determination of total phenolic content

The total phenolic content was determined using the Folin-Ciocalteau reagent (FCR), as previously described (20), with minor modifications. Briefly, 2.5 ml ethanolic mulberry extract was mixed with 0.5 ml FCR (Sigma) and 1.0 ml 20 g/100 g solution of sodium carbonate. The mixture was then incubated for 2 h in the dark at 25°C. The absorbance of the mixture was measured at 765 nm using a UV-Vis Genesys 10 UV spectrophotometer (Thermo Spectronic). A standard curve was plotted using gallic acid (0.07–10 mg/ml in methanol; Sigma) as a standard. The total phenolic content was expressed as gallic acid equivalents (GAEmM/Gfw). The assay was carried out in triplicate and the mean value was recorded.

### Determination of ferric-reducing antioxidant power (FRAP)

FRAP was measured as previously described (21). Briefly, FRAP reagent, which consisted of 0.3 M acetate buffer (pH 3.6), 10 mM 2,4,6-tris(2-pyridyl)-s-triazine (TPTZ) (Fluka, Buchs, Switzerland) in 40 mM HCl and 20 mM FeCl_3_.6H_2_O at a ratio of 10:1:1 (v/v/v) was freshly prepared prior to each measurement. Following this, 200 μl mulberry extract was mixed with 1.3 ml FRAP reagent and incubated for 30 min at 37°C. The absorption was measured at 595 nm using an Epoch spectrophotometer (Bio-Tek Instruments, Inc. Winooski, VT, USA) with the Gen5 Data Analysis Software interface. Aqueous or methanol solutions containing known Fe(II) concentrations were used to calibrate the FRAP assay. FRAP values, expressed as mmol of Fe(II) equivalents (FeFmM/gFW), were determined by comparing the change in the absorption of the test mixture with that of the Fe(II) standards. The assay was carried out in triplicate and the mean value was recorded.

### Evaluation of the anti-diabetic effects of ANCs in ZDF rats

Five-week-old male ZDF (Lepr^fa^/CrlCrlj) and age-matched lean rats (Lepr^fa^/±) were used in this study. All rats were ordered as bred from Charles River Laboratories International (Wilmington, MA, USA). All animal studies were conducted according to the National Institutes of Health Guidelines for the Care and Use of Animals, and were reviewed and approved by the Committee on Animal Experimentation of Kagoshima University (Kagoshima, Japan). The rats were kept under pathogen-free conditions with a 12-h light-dark cycle (lights on at 07:00) at 22±1°C.

The ZDF and lean rats were treated with 125 or 250 mg ANCs/body weight dissolved in 1% CMC (Sigma) in distilled water by gavage, twice daily. The control groups received 1% carboxymethylcellulose (CMC) in distilled water alone.

Following the allocation of the rats to each experimental group, the rats were left to acclimatize and were fed a control diet for 1 week. Food was then withheld for 24 h and tail vein blood samples were collected subsequent to cutting the tip of the tail with a scalpel. The blood samples were centrifuged, and the plasma was stored at −20°C until assay. Blood glucose levels were monitored every week using a glucose meter (Accu-Chek^®^ Active; Roche Diagnostics). Following 5 weeks of treatment with ANCs or CMC, the rats were sacrificed by heart puncture using sterile needles and syringes under anesthesia with diethyl ether, and blood was collected. Plasma insulin levels were measured using an enzyme immunoassay (Cayman Chemical Co., Ann Arbor, MI, USA). All experiments were performed using conscious unrestrained rats.

Following the sacrifice of the rats, the pancreas was perfused with physiological saline and rapidly excised. The tissue samples were maintained in 10% neutral-buffered formalin, dehydrated in a graded ethanol series, cleared in xylene and embedded in paraffin wax. Sections (4 μm thick) were stained with hematoxylin and eosin (H&E). For histological analysis, the tissue sections were photographed using a high-resolution color digital camera mounted on an Olympus BX51 microscope (Olympus, Tokyo, Japan), and the images were transferred to a computer. Four sections were examined from each animal in each treatment group.

### Cell culture and treatment

Murine macrophage-like cells (RAW 264.7) and rat renal tubular epithelial cells (NRK-52E) were obtained from the American Type Culture Collection (Manassas, VA, USA). RAW 264.7 cells were maintained in RPMI-1640 medium (Gibco BRL, Grand Island, NY, USA) supplemented with 10% fetal bovine serum and 2 mmol/l glutamine (Hyclone, Logan, UT, USA). NRK-52E cells were grown in Dulbecco’s modified Eagle’s medium (DMEM; Gibco BRL) containing 7% (v/v%) fetal bovine serum and 2 mmol/l glutamine (Hyclone). RAW 264.7 cells (3.5×10^4^ cells/well) and NRK-52E cells (4×10^4^ cells/well) were cultured in serum-free Opti-MEM^®^ I medium (Gibco BRL) and serum-free DMEM, respectively, prior to stimulation with various concentrations (0, 2, 10, 30, 50 or 100 μg/ml) of mulberry extract for 24 h.

### Methylthiazolyl-diphenyl-tetrazolium bromide (MTT) assay

Cell viability was assessed using a modified MTT assay. Briefly, following the exposure of the cells to the specified concentration of mulberry extract for 48 h, MTT solution was added to each well of the six-well plate. Three hours subsequently, dimethyl sulfoxide (DMSO) was added and the plate was incubated for 24 h at 37°C. Absorbance was measured at 570 nm using an automatic microplate reader (ImmunoMini NJ-2300; InterMed, Tokyo, Japan).

### Statistical analysis

Data were analyzed using SPSS statistical software version 3.0 (SPSS, Inc., Chicago, IL, USA). Data are shown as the mean ± standard deviation. The significance of the differences between two groups was assessed using the Student’s t-test, and differences between multiple groups were assessed by one-way analysis of variance (ANOVA) followed by the Scheffé’s multiple range test. Values of P<0.05 were considered to indicate a statistically significant difference.

## Results

### Analysis of mulberry ANCs

**Figure 1:**
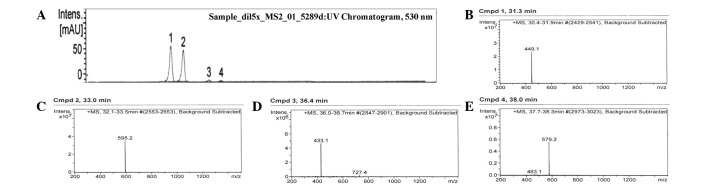
High-performance liquid chromatography (HPLC)-electrospray ionization (ESI)-mass spectometry (MS) analysis of mulberry anthocyanins (ANCs). (A) Chromatogram for the ANC extract of mulberry fruit at 520 nm. Four peaks were detected with retention times ranging from 31 to 38 min. Chromatograms for (B) cyanidin 3-O-glucoside, (C) cyanidin 3-rutinoside, (D) pelargonidin 3-glucoside and (E) pelargonidin 3-rutinoside. The parameters used for peak identification are listed in Table I. Intens, intensity; Cmpd, compound.

The ANC composition of mulberry fruit was determined by HPLC-ESI-MS. The ANC extract was purified using a C-18 Sep-Pak cartridge, and the resulting chromatogram, at 520 nm, is shown in Fig. 1. The chromatogram contained four peaks within the retention time of 31–38 min, indicating the presence of four different ANCs in mulberry fruit (Table I). Peak 1, with a retention time of 31.3 min, M+ at *m/z* 449.1 and a fragment ion at *m/z* 287.0, was identified as cyanidin 3-O-glucoside (51.4%). Peak 2, with a retention time of 33.0 min, M+ at *m/z* 595.2 and fragment ions at *m/z* 449.1 and 287.0, was identified as cyanidin 3-rutinoside (45.3%). Peak 3, with a retention time of 36.4 min, M+ at *m/z* 433.1 and a fragment ion at *m/z* 271.0, was identified as pelargonidin 3-glucoside (2.1%). Peak 4, with a retention time of 38.0 min, M+ at *m/z* 579.1 and fragment ions at *m/z* 433.1 and 271.0, was identified as pelargonidin 3-rutinoside (1.2%). The results of the UV-Vis quantification of the total ANC content showed that the phenolic-rich extract contained 28 mg/g of total ANCs (calculated as cyanidin-3-O-glucoside equivalents). The total phenolic content of ANC extracts, expressed as mmol of Fe(II) equivalents and gallic acid equivalents, was 67.28 GAEmM/Gfw and 22.67 FeFmM/gFW, respectively (data not shown).

### Hypoglycemic effects of ANCs and histology of pancreatic islets in ZDF rats

Table II shows the changes in body weight observed in the six groups of rats. The ZDF rats had significantly higher body weights than their lean littermates from 8 weeks of age, and the body weight progressively increased with age (P<0.05). ANC treatment did not affect body weight in either genotype. Moreover, following 4 weeks of treatment, ZDF rats treated with 250 mg/kg ANCs tended to gain more weight than those treated with CMC alone or with 125 mg/kg ANCs, although this was not statistically significant (P=0.3 versus CMC; P=0.11 versus 125 mg/kg ANCs).

**Figure 2:**
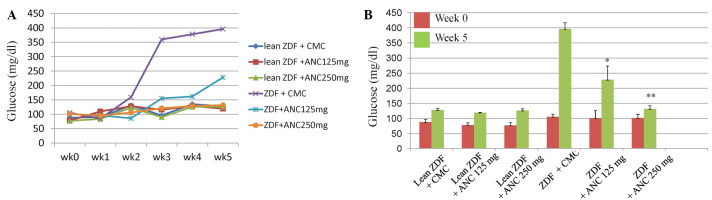
Blood glucose levels of Zucker diabetic fatty (ZDF) and lean ZDF rats treated with 125 or 250 mg/kg anthocyanin (ANC) or 1% carboxymethylcellulose (CMC; vehicle control). (A) Blood glucose levels measured every week during the experimental period. ANCs lowered the glucose levels in the ZDF rats within 3 weeks of treatment in comparison with the levels in the CMC-treated rats. (B) Change in glucose levels from week 0 to week 5. The results are shown as the mean ± standard deviation (n=3–5 rats/group). ^*^P<0.001 and ^**^P<0.0001 vs. CMC-treated ZDF rats.

Blood glucose levels were measured in all of the rats for 5 weeks prior to the commencement of the study and throughout the experimental period (Fig. 2A). At 7 weeks of age, the ZDF rats treated with the vehicle showed mild hyperglycemia (~159 mg/dl) that rapidly progressed, reaching levels of ~396 mg/dl after 3 weeks. The administration of ANCs did not affect the glucose levels in the lean rats. Glucose levels increased significantly from 105.5±8.7 mg/dl at 0 weeks to 396.25±21 mg/dl (P<0.0001) at 5 weeks in the ZDF rats treated with CMC; however, the glucose levels were significantly lower in the rats treated with 125 and 250 mg/kg ANCs (228.25±45 and 131.75±10 mg/dl, respectively; P<0.001 for each; Fig. 2B). Treatment with 250 mg/kg ANCs reduced glucose levels in the ZDF rats to values similar to those in their lean littermates (Fig. 2).

**Figure 3:**
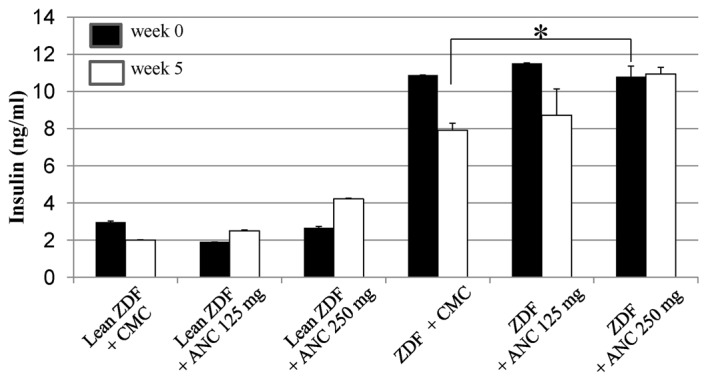
Plasma insulin levels in Zucker diabetic fatty (ZDF) and lean ZDF rats treated with 125 or 250 mg/kg anthocyanin (ANC) or 1% carboxymethylcellulose (CMC) for 5 weeks. Plasma insulin levels at week 0 were significantly higher in the ZDF rats than in their lean littermates (P<0.001). The insulin secretion in the 250 mg/kg ANC-treated ZDF rats at week 5 was 27% higher than that in the CMC-treated ZDF rats. The results are shown as the mean ± standard deviation (n=3–5 rats/group). ^*^P<0.001.

At the start of treatment, when the rats were 5 weeks of age, plasma insulin levels were significantly higher in the ZDF rats than in the lean rats (11±0.2 versus 4.2±0.0 pg/ml; P<0.001; Fig. 3). Between 0 and 5 weeks, the insulin levels decreased from 10.88±0.0 to 7.9±0.4 ng/ml (P<0.05) in the CMC-treated ZDF rats, and from 11.51±0.0 to 8.72±1.4 ng/ml (P<0.05) in the ZDF rats treated with 125 mg/kg ANCs. Notably, plasma insulin levels did not decrease in the ZDF rats treated with 250 mg/kg ANCs (0 weeks: 10.8±0.6 ng/ml; 5 weeks: 10.93±0.4 ng/ml).

**Figure 4:**
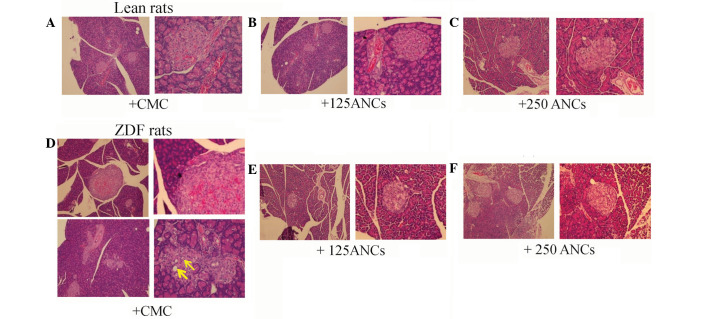
Representative hematoxylin/eosin (H&E)-stained pancreas tissue sections from 11-week-old obese Zucker diabetic fatty (ZDF) and lean rats treated with 125 or 250 mg/ml anthocyanin (ANC) or 1% carboxymethylcellulose (CMC). (A-C) There were no pathological abnormalities in the islets of lean rats. The islets consisted of small, rounded aggregates of mildly eosinophilic cells and were regularly shaped with well-defined boundaries. (D) There were marked morphological changes in the islets of obese ZDF rats, as the islets were hypertrophied and compressed adjacent exocrine tissue, resulting in vascular congestion and hemorrhage. The islets were also disorganized, with extensions into the surrounding exocrine tissue. The degenerated islets showed β-cell vacuolation and degeneration (arrows). (E) Islets of ZDF rats treated with 125 mg/kg ANC showed a normal distribution within the exocrine tissue and mild β-cell vacuolation. (F) There were substantially fewer degenerated islets in ZDF rats treated with 250 mg/kg ANC. The islets in these rats were regularly shaped with well-defined boundaries. H&E staining; original magnification, ×100 (left image) and ×200 (right image).

A histological evaluation of the pancreatic islets of 10-week-old ZDF and lean rats was also conducted. H&E staining revealed no significant pathological abnormalities in the islets from the lean rats, which were round or oval with well-defined boundaries (Fig. 4A–C). However, histological examination of the pancreatic islets from the CMC-treated ZDF rats revealed substantial changes in islet morphology. In particular, the islets were hypertrophic and compressed adjacent exocrine tissue, and there was marked vascular congestion or hemorrhagic degeneration (Fig. 4D, upper panel). Furthermore, the islets were disorganized, with finger-like projections into the surrounding exocrine tissue. The degenerated islets also showed β-cell vacuolation and degeneration (Fig. 4D, lower panel). By contrast, the histological assessment of pancreatic sections from the ZDF rats treated with 125 mg/kg ANCs showed a normal distribution of islets within the exocrine tissue and some β-cell vacuolation (Fig. 4E). Notably, the evaluation of the pancreatic tissue samples collected from the ZDF rats treated with 250 mg/kg ANCs suggested that this dose had certain protective effects, since there were fewer abnormal morphological features and fewer degenerated islets. Additionally, the islets demonstrated a regular shape with well-defined boundaries (Fig. 4F).

**Figure 5:**
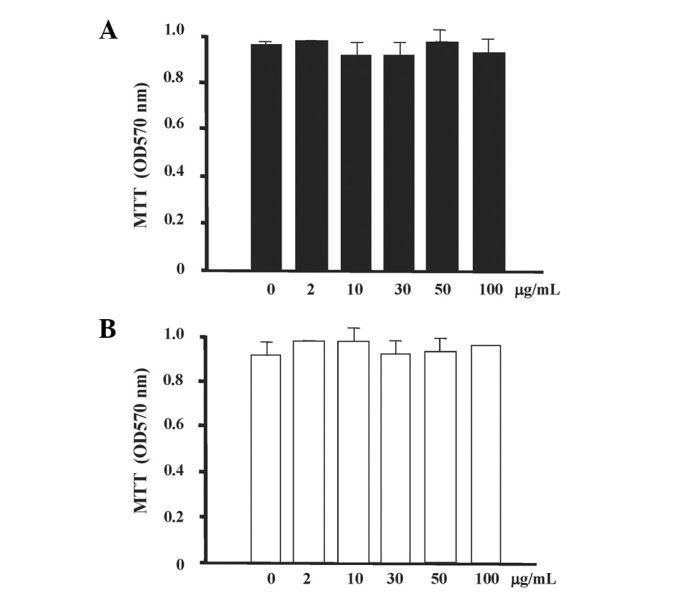
Cytotoxic effect of anthocyanins (ANCs) on (A) murine macrophages and (B) rat kidney cells. Cells were exposed to the indicated concentration of ANC for 48 h and cell viability was quantified using an MTT assay. The results are shown as the mean ± standard deviation for two separate experiments, with each condition performed in duplicate.

In the cell culture studies, it was observed that the ANCs did not exert any cytotoxic effects on the murine macrophages or rat kidney cells (Fig. 5).

## Discussion

The results from this study suggest that ANCs extracted from mulberry fruit exhibit significant anti-diabetic properties by improving blood glucose levels in ZDF rats as an animal model of type 2 diabetes. To the best of our knowledge, this has shown for the first time that ANCs are able to attenuate islet degeneration in ZDF rats. The results demonstrating that ANCs reduced blood glucose levels were consistent with those of a prior study showing that ANCs extracted from black soybean seed coats exhibited antidiabetic and antioxidative effects in streptozotocin-induced diabetic rats (22). Furthermore, the administration of ANCs extracted from *Calendula officinalis* fruits has been demonstrated to significantly increase insulin release from pancreatic β-cells *in vitro*(8).

In the present study, the chromatogram of the purified product following acid hydrolysis of the ethanol extract revealed that cyanidin 3-O-glucoside (51.4%) and cyanidin-3-rutinoside (45.3%) were the major ANCs present in Thai *Morus alba L.* The minor ANCs, which comprised 3.3% of the total ANCs, were pelargonidin 3-O-glucoside and pelargonidin 3-O-rutinoside. These results were consistent with those revealed in the study by Qin *et al*(23), although the ANC content differed, most likely due to differences between mulberry species and cultivars, as well as differences in extraction, separation, purification and analysis between the two studies.

Lean ZDF rats have been demonstrated to be less sensitive to exogenous glucose-induced hyperglycemia (23). The ZDF rats in the present study exhibited marked hyperglycemia at 7 weeks of age and their blood glucose levels continued to increase with age. These results were consistent with those of a previous study in which diabetes occurred spontaneously in male rats aged ~6 weeks, and was associated with hyperphagia, polyuria and polydipsia (24). It was also revealed that the β-cell mass decreased by 51% from 9 to 12 weeks of age (24). In rats aged 6–12 weeks, the β-cell mass is not able compensate for insulin resistance, resulting in compensatory β-cell proliferation (25).

In a previous study, treatment with the ANC cyanidin 3-O-glucoside reduced the body weight and fat accumulation in visceral adipose and liver tissues of KK-Ay mice by improving triglyceride metabolism and regulating lipoprotein lipase activity (26). In another study, aqueous mulberry extract exhibited anti-obesity effects by upregulating hepatic peroxisome proliferator-activated receptor α and carnitine palmitoyltransferase-1 expression, and reducing fatty acid synthase and 3-hydroxy-3-methylglutaryl-coenzyme A (HMG-CoA) reductase expression (14). However, in the present study, the ANC extract from mulberry fruit did not promote reductions in body weight. In fact, the dose of 250 mg/kg ANCs resulted in a certain level of weight gain in the ZDF rats from the age of 9 weeks (P>0.05), without changes in food intake. The differences in results may be due to differences in the polyphenols contained in the extracts or their free radical scavenging properties and mechanisms of action.

To the best of our knowledge, this study has demonstrated for the first time that mulberry fruit extract contains abundant cyanidin 3-O-glucoside (~28 mg/g of crude ANC extract), with the highest ANC dose (250 mg/kg body weight) containing ~7 mg cyanidin 3-O-glucoside. Blood glucose levels were 66% lower and insulin levels were 27% higher in the ZDF rats treated with 250 mg/kg ANCs than in those treated with CMC between 5 and 10 weeks of age. In addition, the consumption of ANCs did not affect glycemia in lean rats. The maximum dose of ANCs used in this study was derived from the cyanidin 3-O-glucoside concentration (10 mg/kg) used in a prior study (11). To date, there are limited data on the mechanisms of ANCs with regard to insulin-mediated glucose uptake. Certain studies have shown that cyanidin 3-O-glucoside from black beans significantly upregulated glucose transport 4 (GLUT4) expression, induced adipocyte differentiation and glucose uptake *in vitro*(27), and prevented insulin resistance and pancreatic apoptosis in streptozotocin-induced diabetic rats (28).

In the present study, the islets of the lean rats showed normal histological features. By contrast, there were marked morphological changes, including islet hypertrophy and cellular degeneration, in the CMC-treated ZDF rats. These pathological observations were consistent with those of earlier studies showing pancreatic islet hypertrophy in ZDF rats (29). By the time diabetes is diagnosed, β-cells attempt to secrete sufficient insulin to overcome the insulin resistance in a process that involves islet hyperplasia. Degenerating islet cells show cytoplasmic vacuolation, possibly resulting from autodigestion following cell death (25). In the present study, the histological assessment of the pancreatic islets from the ZDF rats demonstrated that 250 mg/kg ANCs attenuated the degenerative changes in the majority of the rats. Furthermore, the ANC extract prevented marked reductions in the plasma insulin levels in these rats. These effects may be coupled with enhanced hepatic/peripheral tissue glucose uptake. It was not possible to clarify the mechanism from the current results. Further studies are required to identify the mechanisms of action of ANCs using isolated islets or β-cells to examine whether ANCs have direct effects on insulin secretion.

In conclusion, our results suggest that the ANC extract of mulberry fruit is an effective anti-diabetic agent with marked glucose-lowering effects that prevents the progressive decline in insulin secretion. Although ANCs may protect against β-cell damage, further studies are required to examine the pharmacokinetics and the molecular basis for the pharmacological activity of ANCs on insulin resistance and glucose handling in the management of diabetes mellitus. Long-term studies are required to confirm the present results and to establish the durability of the improvements in glucose levels.

## Figures and Tables

**Table I:** Identification of anthocyanins (ANCs) in mulberry fruit.

Compound number^a^	Retention time (min)	MS, M+ (*m/z*)	MS/MS (*m/z*)	Assignment^b^
1	31.3	449.1	287.0	Cyanidin 3-O-glucoside
2	33.0	595.2	449.1/287.0	Cyanidin 3-rutinoside
3	36.4	433.1	271.0	Pelargonidin 3-glucoside
4	38.0	579.1	433.1/271.0	Pelargonidin 3-rutinoside

a Diode array detection at 350 nm;

b Based on the fragmentation pattern and its aglycone. The assay was performed in triplicate.

MS, mass spectrometry.

**Table II:** Changes in body weight in each experimental group.

	Body weight (g)			
	
Group	0 weeks	2 weeks	4 weeks	5 weeks
Lean rats				
+1% CMC	124±6	152±2	226±6	277±10
+125 ANCs	121±13	153±6	226±4	288±15
+250 ANCs	118±5	149±7	211±11	270±13
ZDF rats				
+1% CMC	143±2	182±4	256±30	324±24
+125 ANCs	139±3	187±6	257±9	317±34
+250 ANCs	140±6	188±6	273±15	331±7

ZDF, Zucker diabetic fatty; +1% CMC, treated with 1% carboxymethylcellulose; +125 ANCs, treated with 125 mg anthocyanins/kg body weight; +250 ANCs, treated with 250 mg anthocyanins/kg body weight.
